# TUBB2A expression and its prognostic significance in hepatocellular carcinoma revealed by cholesterol-metabolism-related gene profiling

**DOI:** 10.3389/fmolb.2026.1778506

**Published:** 2026-03-16

**Authors:** Shiwei Zhu, Yatong Ruan, Mengqi Zhao, Qianqian Zhang, Lulu Zhang, Wen Xu, Hui Zhang, Yuting Qian, Junjie Lin, Ruolin Wu, Ying Dai, Yufeng Gao, Ran Jia, Yuanyuan Wei, Honghai Xu

**Affiliations:** 1 Department of Pathology, The First Affiliated Hospital of Anhui Medical University, Hefei, China; 2 Innovation and Entrepreneurship Laboratory for College Students, Anhui Medical University, Hefei, China; 3 School of Public Health, Anhui Medical University, Hefei, China; 4 Department of Infectious Diseases, The First Affiliated Hospital of Anhui Medical University, Hefei, China; 5 Anhui Province Key Laboratory of Infectious Diseases, Anhui Medical University, Hefei, China; 6 Department of Hepatopancreatobiliary Surgery and Organ Transplantation Center, Department of General Surgery, The First Affiliated Hospital of Anhui Medical University, Hefei, China; 7 Medical Oncology, The First Affiliated Hospital of Anhui Medical University, Hefei, China; 8 Department of Hepatobiliary and Pancreatic Surgery, the First Affiliated Hospital of Anhui Medical University, Hefei, China; 9 Department of Hospital Infection Prevention and Control, The First Affiliated Hospital of Anhui Medical University, Hefei, China

**Keywords:** bioinformatics, cholesterol metabolism, gene set enrichment analysis, hepatocellular carcinoma, medical knowledge–assisted machine learning, prognostic biomarker, single-cellRNA sequencing, TUBB2A

## Abstract

**Introduction:**

Hepatocellular carcinoma (HCC) exhibits significant molecular heterogeneity and is associated with a poor prognosis. The lack of validated biomarkers limits early diagnosis and effective prognosis. Identifying oncogenic drivers in HCC may enhance risk stratification and provide new therapeutic targets. Recent evidence links disrupted cholesterol metabolism to hepatic oncogenesis, and a comprehensive profiling of cholesterol-related genes may help identify metabolic oncogenic signatures and prognostic biomarkers for HCC.

**Methods:**

Bioinformatic analyses were performed using public databases to assess differential expression and the prognostic significance of TUBB2A. Gene set enrichment analysis (GSEA) was conducted to identify key biological pathways associated with TUBB2A expression in HCC. These findings were validated through RT-qPCR, Western blot, and immunohistochemistry on patient tissues. Functional studies included siRNA knockdown and plasmid overexpression in HCC cell lines, followed by assays for cellular proliferation, clonogenicity, migration, and invasion. Tumorigenicity was tested using xenograft models in nude mice. The prognostic value of TUBB2A was evaluated through survival curves and time-dependent ROC analysis.

**Results:**

TUBB2A was identified as a dysregulated and prognostically significant biomarker in HCC through bioinformatic analyses. Pathway analysis using databases like KEGG, GOBP, and Hallmark revealed significant enrichment of TUBB2A in pathways related to cholesterol metabolism, fatty acid biosynthesis, and steroid biosynthesis. Experimental validation demonstrated that TUBB2A is overexpressed in HCC tissues and cell lines. Elevated TUBB2A expression correlated with higher AFP levels, microvascular invasion, advanced tumor stages, and poorer overall survival. Functional assays showed that knockdown of TUBB2A suppressed proliferation, migration, invasion, and *in vivo* tumorigenicity. Furthermore, TUBB2A was found to be associated with key regulators of cholesterol metabolism, including HMGCR, LDLR, SREBP2, and CYP7A1, suggesting its role in regulating cholesterol metabolic homeostasis.

**Discussion:**

TUBB2A plays a key role in promoting HCC tumorigenesis and is associated with adverse clinical outcomes. The integration of bioinformatic analyses and experimental validation establishes TUBB2A as a potential prognostic biomarker in HCC. Its role in regulating cholesterol metabolism suggests that TUBB2A may be a novel target for therapeutic interventions. Further studies should explore the clinical utility of TUBB2A, including its integration into multi-marker models and as a target for targeted therapy, offering potential avenues to improve HCC treatment strategies.

## 1 Introduction

Liver cancer is a significant global public question worldwide, standing at the 6th place for incidence and the 3rd place for mortality among all cancers (Huang et al., 2021; Rumgay et al., 2022). Hepatocellular carcinoma (HCC) is the predominant histological subtype, accounting for approximately 75%–85% (Zhou et al., 2023). Nowadays substantial progress in elucidating the etiological factors contributing to HCC development, including chronic viral hepatitis, excessive alcohol consumption, obesity, and genetic susceptibility (Ge et al., 2023; Singal et al., 2023; Wang et al., 2025). However, HCC continues to impose a significant global burden owing to its high incidence and mortality rates. Moreover, the strong tendency toward recurrence and metastasis further contributes to the unfavorable prognosis of affected patients (Llovet et al., 2021; Forner et al., 2022). There is a critical need to identify and validate new biomarkers that could enhance the early detection of HCC, enable more accurate prognostic stratification, and guide personalized therapeutic strategies, ultimately enhancing treatment efficacy, reducing recurrence risk, and prolonging patient survival.

Cholesterol metabolism is essential for maintaining cell membrane integrity and regulating signal transduction (Lingwood and Simons, 2010; Ng et al., 2022; Christofidi et al., 2025), and is reprogrammed in various malignancies to support uncontrolled cell proliferation (Cheng et al., 2015; Li et al., 2016; Xiao et al., 2023). Consequently, systematic investigation of cholesterol metabolism-related genes may provide novel insights into tumor biology and facilitate the identification of prognostic biomarkers. Against this background, the present study integrated transcriptomic data from the Gene Expression Omnibus (GEO) and The Cancer Genome Atlas (TCGA) databases. Distinct expression analysis was first conducted to identify genes upregulated in tumor tissues. These genes were then intersected with cholesterol metabolism, cell proliferation, and apoptosis-related gene sets from the GeneCards database to screen for candidate genes. Subsequently, a comprehensive analysis pipeline incorporating LASSO regression, random forest, and Cox regression models was applied, resulting in the identification of nine key genes strongly linked with overall survival (OS). Ultimately, TUBB2A expression was notably elevated in HCC tissues and was closely tied to unfavorable prognosis. Single-cell RNA sequencing revealed that TUBB2A was predominantly expressed in hepatocyte and epithelial cell subpopulations. Moreover, time-dependent receiver operating characteristic (ROC) curve provided additional validation for prognostic ability of TUBB2A in predicting long-term survival.

The TUBB2A gene resides on human chromosome 6 and encodes the β-2A tubulin subunit, which constitutes a fundamental structural component of microtubules (Ti et al., 2018). Microtubules are crucial for maintaining cellular structure, enabling intracellular transport and mitosis (Huang et al., 2022; Jansen et al., 2023; Volkov and Akhmanova, 2024). Pathogenic variants in TUBB2A have been shown to disrupt microtubule dynamics, resulting in a spectrum of neurodevelopmental abnormalities, including brain structural malformations and impaired neuronal migration. Clinically, these defects manifest as developmental delay, intellectual disability, and epilepsy (Cai et al., 2020; Brock et al., 2021; Di Pasquale et al., 2025). Consistent with its role in microtubule regulation, gene set enrichment analyses have demonstrated that microtubule-associated pathways involving tubulin family members, such as TUBB2A, are significantly enriched in transcriptomic signatures of neurological disorders, including Parkinson’s disease and Huntington’s disease (Fan et al., 2025; Shin et al., 2025). Beyond the nervous system, accumulating evidence indicates that aberrant TUBB2A expression is also implicated in tumorigenesis. Elevated TUBB2A levels have been reported in various malignancies, including non-small cell lung cancer,breast cancer, and prostate cancer, where they are linked to aggressive tumor behavior and adverse clinical outcomes (Haram et al., 2008; Joerger et al., 2011; Li et al., 2024).

Nevertheless, the function of TUBB2A in hepatocellular carcinoma remains largely undefined. To initiate our investigation, we first identified TUBB2A as a markedly upregulated gene in HCC tissues through integrative bioinformatics analysis, with its expression closely linked to patient prognosis. We subsequently performed comprehensive experimental validation to confirm its aberrant expression, prognostic relevance, and functional effects on malignant phenotypes of HCC cells. In addition, we explored the potential link between TUBB2A and cholesterol metabolism by measuring the expression of key regulators of cholesterol homeostasis, including HMGCR, LDLR, SREBP2, and CYP7A1 (Saito et al., 2023; Xiao et al., 2023; Cao and Liu, 2023; Chan et al., 2026). Collectively, these findings provide a theoretical basis and suggest TUBB2A as a promising biomarker for evaluating prognosis and facilitating the diagnosis of HCC.

## 2 Materials and methods

### 2.1 Tissue samples

Clinicopathological data from 121 HCC patients treated in the Department of Hepatobiliary Surgery, between January 2019 and March 2020 were collected. We also obtained paraffin-embedded tumor samples along with matched adjacent non-neoplastic tissues. Additionally, we gathered seven fresh HCC samples along with their matched adjacent non-cancerous samples. The inclusion criteria were defined: (1) postoperative pathological examination confirmed the diagnosis of HCC; (2) complete medical records; (3) no systemic antitumor therapy. Follow-up data were complete for all cases, and as of October 2024, OS was measured as the period from HCC diagnosis to either patient death or the last follow-up visit. This study was approved by the Ethics Committee of the First Affiliated Hospital of Anhui Medical University (PJ 2025-8-42).

### 2.2 Bioinformatics analysis

#### 2.2.1 Data collection, preprocessing, and differential expression analysis

We processed transcriptomic data retrieved from the GEO and TCGA databases using the linear models for microarray data (limma) approach to detect differentially expressed genes. The cutoff value was established at an adjusted *P* < 0.05 and |log_2_FC| > 1. Furthermore, We generated volcano plots to illustrate the overall distribution and expression trends of the differentially expressed genes.

#### 2.2.2 Feature selection and machine learning model construction

To identify essential feature genes from the candidate set, LASSO regression and random forest models were employed for integrated analysis. LASSO regression was conducted using the package (v4.1.8), applying L1 regularization to reduce redundant variables and alleviate multicollinearity. We employed ten-fold cross-validation to pinpoint the optimal penalty parameter λ. The Random Forest package (v4.7.1.2) was employed to assess the importance of candidate genes, thereby identifying key genes with relatively higher contributions to classification tasks.

#### 2.2.3 Prognostic correlation analysis

To quantify the impact of core genes on OS, univariate Cox regression analysis was applied using the Survival package (v3.8.3) with expression data of target genes and clinical covariates from the TCGA-LIHC cohort. The criteria for selection were *P* < 0.05 and HR > 1. Kaplan-Meier (KM) survival analysis was then carried out for the chosen genes. The subjects were grouped in line with the median expression intensity of the target genes, and their survival differences were assessed through the log-rank test. *P*-values below 0.05 were considered indicative of statistical significance.

#### 2.2.4 Single-cell expression analysis

To elucidate how the target genes are expressed across heterogeneous cell populations, we leveraged the Seurat package for the processing and subsequent examination of single-cell RNA sequencing data (v5). Expression profiles across various cell populations were visualized using the FeaturePlot and Violin plot functions, providing an intuitive display of their differential distribution among cell types.

#### 2.2.5 Construction of prognostic model

To further validate the prognostic ability of TUBB2A, a time-dependent ROC curve model was constructed based on the TCGA-LIHC cohort to evaluate its predictive accuracy for OS. Using Time ROC package (v0.4) and Survminer packages (v0.5.0), we quantified AUC values and analyzed the survival predictive ability of TUBB2A at various follow-up times.

#### 2.2.6 Differential expression and enrichment analysis of TUBB2A in TCGA-LIHC dataset

Within the TCGA-LIHC dataset, we selected tumor samples, and defined the high and low expression groups according to the median expression of TUBB2A (Log_2_ (TPM+1)). Differential gene-expression profiling was performed using DESeq2 with raw counts, with thresholds of |Log_2_FC| > 1 and adjusted *P* value <0.05 to identify significantly differentially expressed genes. The resulting genes were sorted according to Log_2_FC, followed by Gene Set Enrichment Analysis (GSEA) using the Gene Ontology Biological Processes (GOBP), KEGG, Reactome, and Hallmark databases. We adjusted the *P*-values for multiple testing using the Benjamini-Hochberg (BH) method.

### 2.3 Experimental methods

#### 2.3.1 Cell transient transfection

Following cryopreservation recovery, we seeded and cultured the normal hepatic cell line MIHA and HCC cell lines (LM3, Huh7, PLC, Hep3B, and MHCC-97H) with complete DMEM medium. We incubated the cell cultures under standard conditions. To quantify TUBB2A expression at the transcriptional and translational levels, we performed RT-qPCR and WB experiments on each cell line, respectively, which helped determine the expression profiles of TUBB2A, and the cell lines to be used in subsequent experiments were selected accordingly. SiTUBB2A and TUBB2A overexpression plasmids were transfected into HCC cells to establish transient transfection models, and shTUBB2A transfection was used to construct stable knockdown models. After we selected the cell lines for follow-up experiments, we measured the mRNA levels of HMGCR, LDLR, SREBP2 and CYP7A1 by RT-qPCR using the same protocol as for TUBB2A, and we used GAPDH as the internal control.

#### 2.3.2 Immunohistochemical (IHC) staining

Following sectioning and drying, we subjected the paraffin-embedded tissue slices to sequential dewaxing with xylene and stained them with hematoxylin for 6 min, followed by dehydration and mounting with neutral resin. Following sectioning and drying, paraffin-embedded tissue slices were subjected to sequential dewaxing with xylene, stepwise rehydration using a graded ethanol gradient (from high to low concentration), followed by standard antigen retrieval procedures to expose target epitopes. The primary antibody against TUBB2A (Novus, catalog number NBP3-16484-100 μL) was diluted following the manufacturer’s guidelines and applied to tissue sections, which were incubated 2 h. Then sections were subjected to three washes using Tris-buffered saline, with each wash performed for 5 min. Afterward, the secondary antibody (catalog number KIT-5010, Maixin) was then applied and incubated for 30 min. Followed by hematoxylin counterstaining, dehydration via a graded ethanol series, and permanent mounting with neutral balsam. Two experienced clinical pathologists evaluated the IHC staining results independently. The staining intensity was scored on a 4-point scale as follows: 0 (no staining), 1 (weak staining), 2 (moderate staining), and 3 (strong staining). The extent of staining was scored based on the percentage of positive cells: 0 (<5%), 1 (5%–25%), 2 (26%–50%), 3 (51%–75%), or 4 (76%–100%) (Fedchenko and Reifenrath, 2014; Meyerholz and Beck, 2018). The total IHC score was derived by multiplying the two together. The score <6 was considered low expression of TUBB2A, and ≥6 was considered high expression of TUBB2A.

#### 2.3.3 WB

Samples from each experimental group were gathered, and an equal volume of RIPA lysis buffer to extract protein, adjusting loading amount accordingly. GAPDH antibody (catalog number 60004-1-Ig, Proteintech) served as an internal reference for normalization. An identical quantity of protein was loaded per sample, separated via electrophoresis, transferred onto membrane, Once the detection reagents were applied and the signal developed, the resulting protein band intensities were visualized and recorded using a chemiluminescence imaging system. Uncropped and original WB images are provided in the Supplementary Material.

#### 2.3.4 RT-qPCR

The RNA was purified from normal liver and HCC samples, and cultured cells by a commercial RNA extraction kit. Following RNA quality verification, 1 μg of qualified RNA was reverse-transcribed, followed by PCR amplification. Relative quantification of TUBB2A expression in HCC tissue and cells were calculated using the 2^(-△△CT)^ method.

#### 2.3.5 CCK-8

The transfected cells were processed, then centrifuged for 4 min, resuspended, then placed into a 96-well plate with 4,000 cells per well. After culturing for 24, 48, 72, and 96 h, 100 μL of culture medium 10% CCK-8 solution was dispensed. Optical density (OD) was subsequently quantified at a wavelength of 450 nm.

#### 2.3.6 Colony formation assay

We treated transfected cells with enzymatic digestion, and centrifuged them at 800 r/min for 4 min. The cell pellet was resuspended to adjust the density to 10 × 10^3^ cells/mL. These cells were then incubated for 2 weeks. Once cell colonies became observable under a light microscope, the culture process was terminated. Next, the cells were rinsed with running water to remove non-specific background coloration. After air-drying, colonies consisting of over 10 cells were counted under the microscope, and photographic records were taken for long-term documentation.

#### 2.3.7 Transwell migration and invasion assays

A Transwell chamber was positioned in a 24-well culture plate, into the upper chamber, cell suspension prepared was added. The assembly was then incubated for 24 h, and the experiment was terminated. After removal of the culture medium, non-migrated cells adhering to the interior were wiped away. We stained cells with crystal violet solution, subsequently rinsed with distilled water, and allowed to air-dry. The stained cells were visualized, and the total migrated cells were quantified.

#### 2.3.8 Scratch assay

Cells were plated in a 6-well culture plate in an optimal initial density. Six hours after transfection, the pipette tip was employed to generate scratches on cells, after which the original culture medium was substituted with serum-free medium. This moment was designated as the 0-h time point. Observations were then taken at 0 h, 12 h, and 24 h time points utilizing an inverted light microscope, with images captured at each interval.

#### 2.3.9 Subcutaneous tumorigenesis assay

Ten 5-week-old male nude mice were assigned randomly to two distinct experimental groups. Cells transfected with shTUBB2A alongside control cells were combined with an equal volume of Matrigel to prepare a suspension. Mice belonging to each of the two groups were administered a 100 µL subcutaneous injection of the suspension at the right lower abdominal site. The mice’s body weight, tumor volume, and general health status were documented on a weekly basis. We calculated tumor volume according to the following formula: V = 1/2×length × width^2^ (Jensen et al., 2008; Li et al., 2016), and a growth curve was plotted based on changes in tumor volume. Following a 4-week experimental period, mice were euthanized by cervical dislocation (Su et al., 2025). Tumors were then photographed, weighed, and fixed in formalin for immunohistochemical analysis.

All animal procedures were approved by the Animal Ethics Committee of Anhui Medical University (Approval No. LLSC20200965) and were conducted in accordance with institutional guidelines and the AVMA Guidelines for the Euthanasia of Animals (2020).

### 2.4 Statistical analysis

All experiments were replicated in triplicate. Statistical analyses were used the IBM SPSS Statistics. The χ^2^ test was used to compare differences between groups. We generated KM survival curves and evaluated differences in cumulative survival among groups with distinct TUBB2A expression levels using the log-rank test. We defined a *P*-value <0.05 as statistically significant.

## 3 Results

### 3.1 Differential expression and feature selection to identify core candidate genes

**FIGURE 1:**
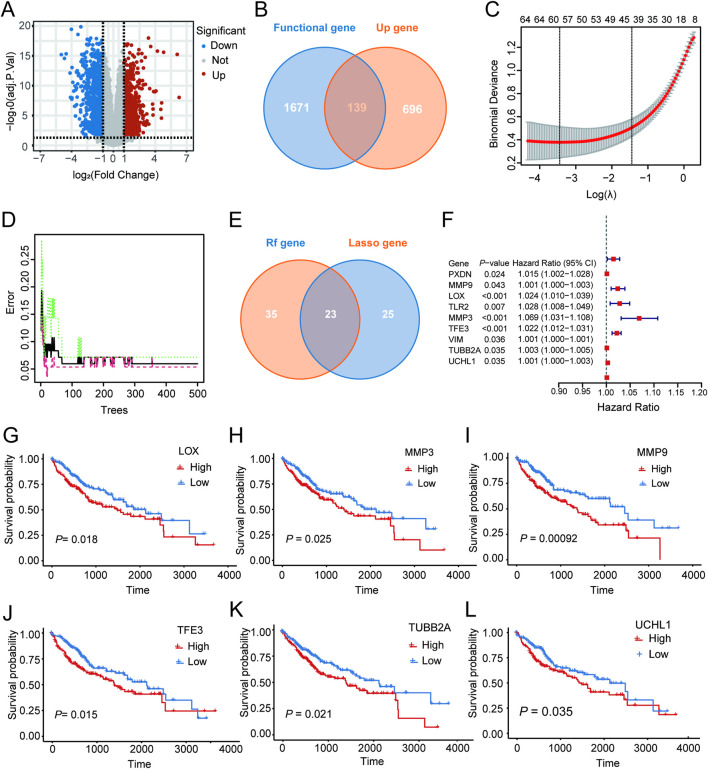
Overall workflow and results of key gene screening and survival analysis. **(A)** Volcano plot of differentially expressed genes in GSE166163. **(B)** Venn diagram showing the intersection between function-related genes from GeneCards (cholesterol metabolism, cell proliferation, and apoptosis) and upregulated genes (n = 139). **(C)** Cross-validation plot of the LASSO regression model used to determine the optimal λ value and select feature genes. **(D)** Variable importance ranking of candidate genes generated using the random forest model. **(E)** Twenty-three core genes obtained from the intersection of LASSO and random forest results. **(F)** Univariate Cox regression analysis based on TCGA-LIHC identifying nine genes significantly associated with overall survival (OS). **(G–L)** Kaplan-Meier survival curves of six representative genes (LOX, MMP3, MMP9, TFE3, TUBB2A, and UCHL1).

The dataset GSE166163 related to HCC was retrieved from the GEO, and differential expression analysis was performed utilizing the limma, with significance thresholds set to *P* < 0.05 and |log_2_FC| > 1. A volcano plot was then created to illustrate the results visually (Figure 1A). Genes related to cholesterol metabolism, cell proliferation, and apoptosis were extracted from the GeneCards database (Supplementary Table). And then intersected with tumor-derived upregulated differentially expressed genes to identify candidate key genes (Figure 1B). LASSO regression and random forest were used for feature selection; LASSO, with L1 regularization and ten-fold cross-validation, determined the optimal λ (approximately 1.58) and identified 48 genes with non-zero coefficients (Figure 1C), while RF identified 58 highly important genes (Figure 1D), and their intersection yielded 23 core genes (Figure 1E). Using TPM and OS data from the TCGA-LIHC cohort, a univariate Cox regression analysis (*P* < 0.05) identified nine genes significantly linked to OS: PXDN, MMP9, LOX, TLR2, MMP3, TFE3, VIM, TUBB2A, and UCHL1 (Figure 1F).

### 3.2 Validation of survival and single-cell localization to highlight the key gene TUBB2A

**FIGURE 2:**
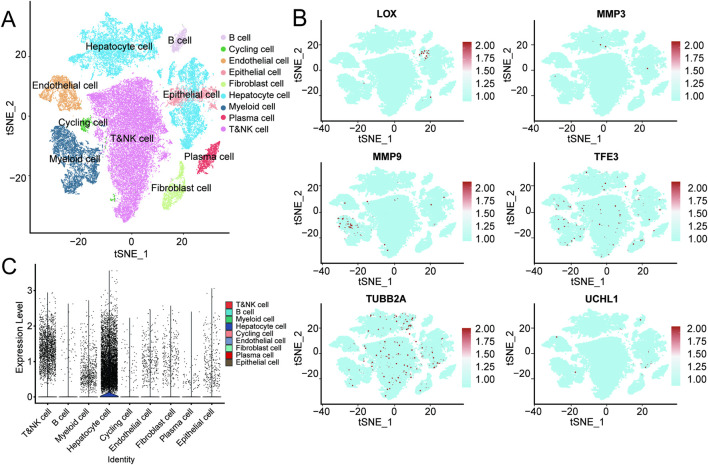
Expression characteristics of key genes in the GSE151530 single-cell atlas. **(A)** t-SNE plot displaying single-cell clusters and annotation of major cell types. **(B)** Feature plots showing the distribution of candidate genes (LOX, MMP3, MMP9, TFE3, TUBB2A, and UCHL1) across different cell subpopulations. **(C)** Violin plots illustrating differential expression of TUBB2A between HCC and normal tissues across various cell types.

To further verify the potential role of the candidate genes in patient survival prognosis, KM survival analysis was conducted. Patients were stratified based on the median expression levels of the target genes, and intergroup survival differences were compared via the log-rank test. Results from the analysis showed that patients with high levels of LOX, MMP3, MMP9, TFE3, TUBB2A, and UCHL1 had significantly reduced survival rates (*P* < 0.05, Figures 1G–L). To examine their expression characteristics across different cell types, the single-cell dataset GSE151530 was analyzed and annotated (Figure 2A). FeaturePlot and ViolinPlot results indicated that TUBB2A was enriched in hepatocytes but showed low expression levels overall in immune cell populations (Figures 2B,C), indicating a distinct cell type-specific expression pattern. Based on this, TUBB2A was included in subsequent prognostic analyses to further assess its relationship with HCC clinical outcomes.

### 3.3 Time-dependent ROC evaluation: prognostic predictive performance of TUBB2A

To further assess the prognostic predictive efficacy of TUBB2A at different follow-up time points, time-dependent ROC analysis was conducted based on the TCGA cohort. In the TCGA cohort, TUBB2A expression was markedly elevated in HCC relative to adjacent normal liver tissues, consistent with the differential expression patterns observed in GEO datasets and clinical samples (Supplementary Figure S1A). The subsequent analysis involved plotting time-dependent ROC curves to evaluate the ability of TUBB2A in forecasting 1-year, 3-year, and 5-year OS outcomes. The AUC values corresponding to 1-year, 3-year, and 5-year OS prediction were 0.587, 0.591, and 0.600, showing a modest but gradual increase over time (Supplementary Figure S1B). These findings indicate that TUBB2A alone exhibits moderate discriminatory ability for survival prediction. The relatively stable AUC values across different time points suggest that the prognostic relevance of TUBB2A is maintained during long-term follow-up rather than being limited to early survival outcomes.

### 3.4 Differential expression and functional enrichment associated with TUBB2A expression

In the TCGA-LIHC dataset, patients were stratified into high- and low-expression groups based on the median TUBB2A expression level (Log_2_ (TPM +1)). Differentially expressed genes (DEGs) were identified using DESeq2 with thresholds of |Log_2_FC| > 1 and adjusted *P* value <0.05. A volcano plot (Supplementary Figure S1C) visually presents the distribution of these DEGs, highlighting significant upregulation in the high-expression group.

For further insight, we performed GSEA to identify enriched biological pathways associated with high TUBB2A expression. KEGG and GOBP pathway analysis revealed significant enrichment in pathways related to cholesterol metabolism, fatty acid degradation, and steroid biosynthesis (Supplementary Figures S1D,E). Furthermore, GOBP GSEA revealed enrichment in metabolic processes, including fatty acid metabolic processes, amino acid metabolic processes, and xenobiotic metabolic processes (Supplementary Figure S1F), underscoring the metabolic alterations driven by high TUBB2A expression in HCC. These pathways indicate that elevated TUBB2A expression contributes to lipid metabolic reprogramming, a key feature of HCC progression. Additionally, Reactome pathway analysis identified enrichment in the cell cycle, DNA replication, and the PI3K-Akt signaling pathway (Supplementary Figure S1G), suggesting that TUBB2A may promote tumor cell proliferation and survival through these oncogenic pathways.

Moreover, Hallmark GSEA showed significant enrichment in pathways related to cell cycle regulation, such as G2/M checkpoint, E2F targets, and MYC targets, further confirming the role of TUBB2A in promoting tumor cell cycle acceleration and growth (Supplementary Figure S1H).

### 3.5 TUBB2A expression levels are elevated in hepatocellular carcinoma tissues relative to adjacent normal liver tissues

**FIGURE 3:**
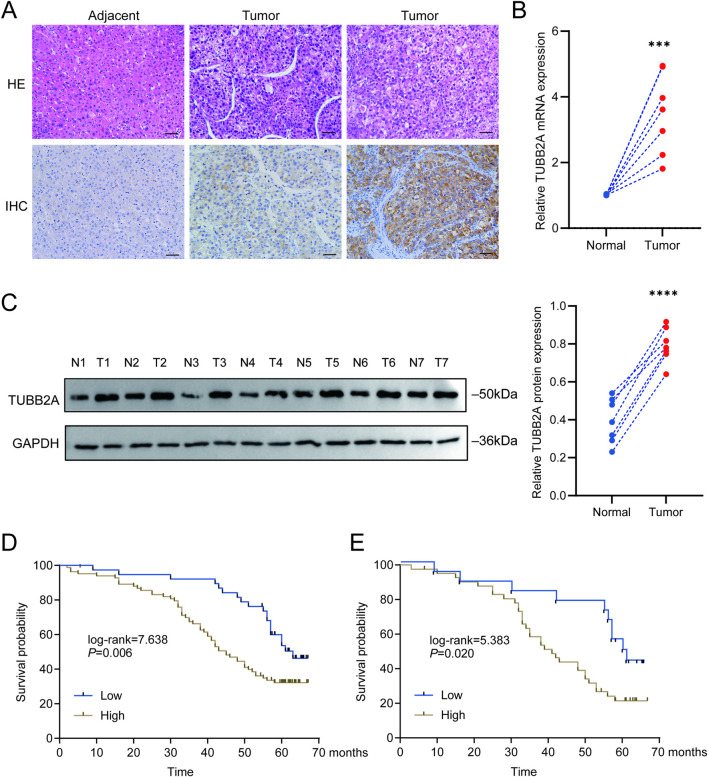
Expression of TUBB2A in HCC tissues and its prognostic significance. **(A)** Above: HE staining showing tissue morphology; below: immunohistochemical staining showing TUBB2A expression in the cytoplasm of hepatocellular carcinoma tissues, with higher expression in tumor tissues compared to adjacent normal tissues. Left: adjacent normal tissue with low TUBB2A expression, middle: HCC with lower TUBB2A expression, right: HCC with higher TUBB2A expression (magnification ×200). **(B)** RT-qPCR analysis of TUBB2A expression in normal and HCC tissues. **(C)** Western blot bands and quantitative analysis of TUBB2A expression in normal and tumor tissues (N: normal; T: tumor). **(D)** Kaplan-Meier survival curves of HCC patients stratified by low versus high TUBB2A expression (n = 121). **(E)** Kaplan–Meier survival curves of HCC patients with tumor diameter ≥5 cm, stratified by TUBB2A expression level (n = 59). Note: ***P ≤ 0.001; ****P ≤ 0.0001.

IHC staining assays performed on a cohort of 121 HCC cases demonstrated that TUBB2A protein was localized within the cytoplasm of tumor cells. Among these cases, high TUBB2A expression was observed in 83 tumor samples, whereas low expression was detected in 38 cases. Overall, the level of TUBB2A protein was elevated in HCC samples compared with their corresponding liver tissues (Figure 3A). Consistent with the IHC findings, RT-qPCR assays performed on 7 paired tissue samples revealed that TUBB2A mRNA levels were upregulated in HCC samples relative to their corresponding adjacent non-tumorous liver tissues (t = 5.333, *P* = 0.0002; Figure 3B). WB analysis further verified a marked upregulation of TUBB2A protein expression (t = 7.029, *P* < 0.0001; Figure 3C).

### 3.6 High TUBB2A expression indicates poor prognosis

Among the 121 included patients with HCC, cases were stratified according to TUBB2A expression level, and χ^2^ test analysis was performed for clinicopathological feature. The results demonstrated that the proportions of patients with alpha-fetoprotein (AFP) ≥ 56.3 ng/mL, microvascular invasion, and pathological stage III–IV were higher in the TUBB2A high-expression group (all *P* < 0.05, Table 1). Surveillance duration for cohort ranged from 2 to 67.13 months, with a median surveillance period of 53 months. Throughout the monitoring phase, we documented a total of 75 deaths and determined the median OS to be 53 months (95% CI: 47.31–58.69 months). Log-rank test results showed that, in both the overall cohort of 121 HCC patients and in the subgroup with tumor diameter ≥5 cm, the cumulative survival rate was notably reduced in the TUBB2A high-expression group (*P* = 0.006; *P* = 0.020; Figures 3D,E).

**TABLE 1:** Association between TUBB2A expression and clinicopathological characteristics in HCC patients.

Clinical Characteristics	TUBB2A low expression (n = 38)	TUBB2A high expression (n = 83)	χ^2^ value	P value
Sex			0.001	0.989
Male	33 (86.8)	72 (86.7)		
Female	5 (13.2)	11 (13.3)		
Age			0.982	0.322
<60 years old	22 (57.9)	40 (48.2)		
≥60 years old	16 (42.1)	43 (51.8)		
Hepatitis B virus			3.234	0.072
No	6 (15.8)	26 (31.3)		
Yes	32 (84.2)	57 (68.7)		
Cirrhosis			0.125	0.724
No	21 (55.3)	43 (51.8)		
Yes	17 (44.7)	40 (48.2)		
AFP(ng/mL)			5.818	0.016*
<56.3	25 (65.8)	35 (42.2)		
≥56.3	13 (34.2)	48 (57.8)		
Tumor number			1.349	0.245
Single	36 (94.7)	71 (85.5)		
Multiple	2 (5.3)	12 (14.5)		
Tumor size			0.043	0.836
<5 cm	20 (52.6)	42 (50.6)		
≥5 cm	18 (47.4)	41 (49.4)		
Microvascular invasion			6.203	0.045*
M0	22 (57.8)	47 (56.6)		
M1	8 (21.1)	30 (36.1)		
M2	8 (21.1)	6 (7.2)		
Satellite nodules			0.178	0.673
Absent	27 (71.1)	62 (74.7)		
Present	11 (28.9)	21 (25.3)		
Capsular invasion			2.903	0.088
Absent	19 (50.0)	55 (66.3)		
Present	19 (50.0)	28 (33.7)		
Degree of differentiation			0.181	0.913
Well differentiated	6 (15.8)	13 (15.7)		
Moderately differentiated	28 (73.7)	59 (71.1)		
Poorly differentiated	4 (10.5)	11 (13.3)		
Pathological stage			4.043	0.044*
I + II	36 (94.7)	67 (80.7)		
III + IV	2 (5.3)	16 (19.3)		

(1) The bold values indicate statistically significant results (*P* < 0.05). (2) The diagnostic cutoff value of 56.3 for AFP, corresponds to the median in this cohort; (3) M0: no microvascular invasion detected; M1: ≤5 foci of microvascular invasion located in the peritumoral liver tissue (≤1 cm from the tumor margin); M2: >5 foci of microvascular invasion and/or microvascular invasion present in distant peritumoral liver tissue (>1 cm from the tumor margin) (Zhou et al., 2023).

### 3.7 Knockdown of TUBB2A inhibits the proliferation, migration, and invasion of HCC cells

**FIGURE 4:**
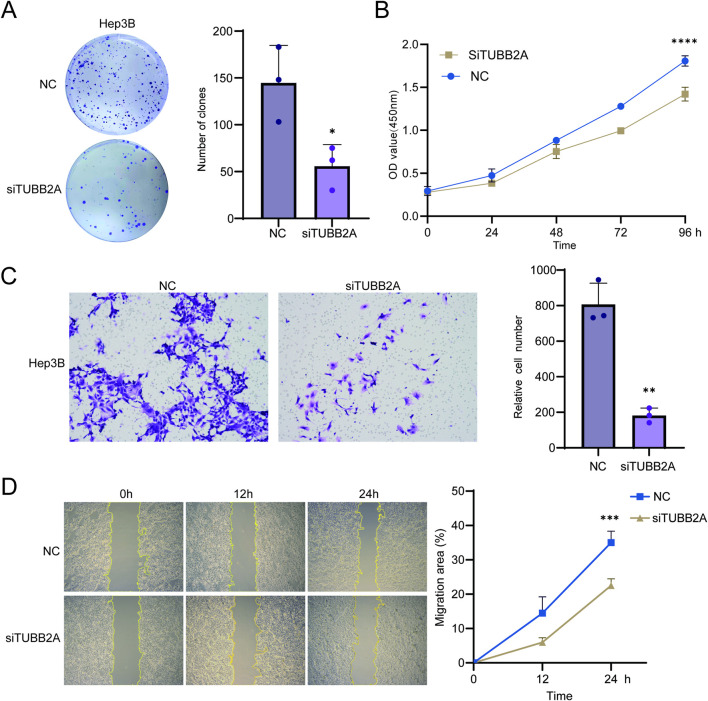
Effects of TUBB2A knockdown on the malignant behavior of HCC cells. **(A)** Effects of TUBB2A knockdown on the clonogenic capacity of Hep3B cells. **(B)** Effects of TUBB2A knockdown on Hep3B cell viability as measured by the CCK-8 assa. **(C)** Effects of TUBB2A knockdown on the migratory and invasive abilities of Hep3B cells, as shown by representative images and quantitative analysis of cell numbers. **(D)** Representative images and quantitative analysis of scratch-wound healing in Hep3B cells following TUBB2A knockdown. Note: NC group, negative control; si group, TUBB2A knockdown. *P ≤ 0.05; **P ≤ 0.01; ***P ≤ 0.001; ****P ≤ 0.0001.

RT-qPCR and WB assays were employed to determine the mRNA and protein expression levels of TUBB2A, respectively, in the cell lines, namely Huh7, LM3, PLC, Hep3B, and MHCC-97H. The results showed that among these HCC cell lines, LM3 had the lowest expression level, whereas Hep3B had the highest (Supplementary Figures S2A,B). Therefore, LM3 was selected to construct the overexpression model, whereas Hep3B was chosen for gene knockdown experiments. In Hep3B cells, transfection with siTUBB2A led to a marked reduction in TUBB2A mRNA as shown by RT-qPCR, and WB simultaneously confirmed an effective downregulation of TUBB2A protein levels (both *P* < 0.05, Supplementary Figure S3A,B). Functional assays demonstrated that colony formation and CCK-8 results indicated a significant suppression of cell proliferative activity and clonogenic capacity (t = 3.329, t = 8.725, *P* = 0.0291, *P* < 0.0001, Figures 4A,B). Transwell assays display a marked reduction in the migratory and invasive capacities for the treated cells (t = 8.543, *P* = 0.0010, Figure 4C). Scratch-wound healing assays further confirmed a decrease in the migration area (t = 6.009, *P* = 0.0002, Figure 4D). Taken together, siTUBB2A achieved effective knockdown of TUBB2A in Hep3B cells and consistently attenuated their proliferative, clonogenic, migratory, and invasive abilities.

### 3.8 Overexpression of TUBB2A promotes the proliferation, migration, and invasion of HCC cells

**FIGURE 5:**
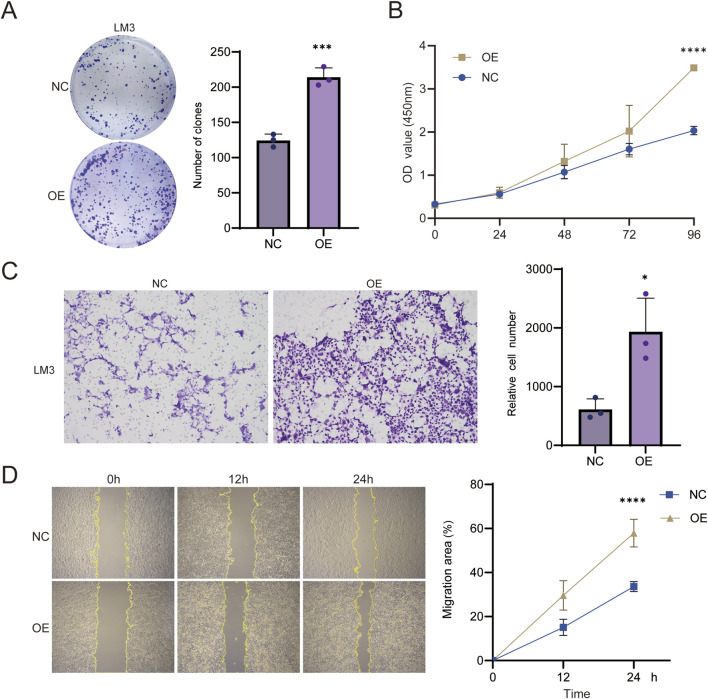
Effects of TUBB2A overexpression on the malignant phenotypes of HCC cells. **(A)** Effects of TUBB2A overexpression on the clonogenic capacity of LM3 cells. **(B)** Effects of TUBB2A overexpression on LM3 cell viability as measured by the CCK-8 assay. **(C)** Representative images and quantification of cell numbers showing the effects of TUBB2A overexpression on migration and invasion in LM3 cells. **(D)** Representative images and quantitative analysis of scratch-wound healing in LM3 cells following TUBB2A overexpression. Note: NC group, negative control group; OE group, TUBB2A overexpression group. *P ≤ 0.05; **P ≤ 0.01. ***P ≤ 0.001; ****P ≤ 0.0001.

LM3 cells were transfected with TUBB2A overexpression plasmids. Subsequent verification via RT-qPCR and WB confirmed that TUBB2A was significantly upregulated in these transfected cells (both *P* < 0.05, Supplementary Figures S4A,B). Colony formation and CCK-8 results demonstrated that cell proliferative capacity and clonogenic potential were markedly enhanced (t = 7.351, t = 9.589, *P* < 0.0001, *P* = 0.0007, Figures 5A,B). Further Transwell experiments revealed a notable increase in the migratory and invasive potential of the treated cell population (t = 3.804, *P* = 0.0190, Figure 5C). Scratch-wound healing assays also showed accelerated wound closure and enhanced migratory capacity (t = 7.187, *P* < 0.0001, Figure 5D). Taken together, overexpression of TUBB2A promotes the proliferation, clonogenic growth, and migratory and invasive abilities of HCC cells.

### 3.9 Knockdown of TUBB2A suppresses tumor growth *in vivo*

**FIGURE 6:**
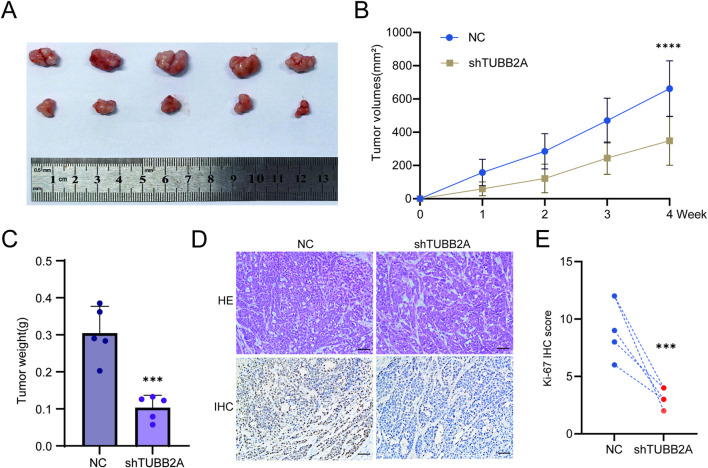
Effects of TUBB2A on the *in vivo* tumorigenic ability of HCC cells. **(A)** Representative images of subcutaneous tumors formed in nude mice in the two groups. Above: Subcutaneous tumors of the control group; below: Subcutaneous tumors of the shTUBB2A group. **(B)** Growth curves showing changes in subcutaneous tumor volume in the two groups of nude mice. **(C)** Comparison of subcutaneous tumor weight between the two groups of nude mice. **(D)** Above: HE staining showing tissue morphology; below: IHC Ki-67 staining. Left: TUBB2A control group nude mouse tumor with low Ki-67 expression; right: TUBB2A knockdown group nude mouse tumor with high Ki-67 expression (magnification ×200). **(E)** Quantification of Ki-67 IHC score in the subcutaneous tumors. Note: ***P ≤ 0.001; ****P ≤ 0.0001.

To confirm the knockdown efficiency, we transduced Hep3B cells with shTUBB2A and measured TUBB2A mRNA and protein levels by RT-qPCR and Western blot, respectively. The results showed a significant decrease in TUBB2A levels in the knockdown group (both *P* < 0.05, Supplementary Figures S5A,B). To investigate the regulatory function of TUBB2A in tumorigenesis *in vivo*, we administered HCC cells with stable shTUBB2A and matched control cells via subcutaneous injection into nude mice. Our data revealed a marked reduction in both tumor volume and tumor weight in mice bearing TUBB2A-knockdown HCC cells, as compared with the control cohort (t = 4.851, *P* < 0.0001; t = 5.666, *P* = 0.0005; Figures 6A-C). Furthermore, IHC staining for Ki-67 in excised tumor tissues demonstrated markedly weaker staining intensity and a significantly reduced percentage of Ki-67-positive cells was observed in the TUBB2A depletion samples compared with corresponding control cohort (Figure 6D). Quantitative analysis further showed that the Ki-67 IHC score differed significantly between the two groups (t = 5.062, *P* = 0.0010, Figure 6E). Collectively, these findings indicate that TUBB2A knockdown significantly suppresses HCC tumor growth and cell proliferation *in vivo*.

### 3.10 TUBB2A expression inversely correlates with cholesterol homeostasis–related gene expression in HCC cells

Based on the GSEA results showing significant enrichment of cholesterol metabolism-related processes associated with TUBB2A, we further validated the relationship between TUBB2A and key regulators of cholesterol homeostasis at the cellular level. We established a TUBB2A overexpression model in LM3 cells and generated a stable TUBB2A knockdown model in Hep3B cells. We then used RT-qPCR to measure the mRNA expression levels of HMGCR, LDLR, SREBP2 and CYP7A1, with GAPDH serving as the internal control. Compared with the control group, they showed an overall upward trend in Hep3B cells with TUBB2A knockdown (all *P* < 0.05, Supplementary Figure S6A); in contrast, these cholesterol homeostasis-related genes showed an overall downward trend in LM3 cells overexpressing TUBB2A (all *P* < 0.05, Supplementary Figure S6B). These findings are consistent with the bioinformatic analysis indicating that high TUBB2A expression is accompanied by altered cholesterol metabolism-related processes, suggesting that TUBB2A may participate in regulating cholesterol metabolic homeostasis in HCC cells.

## 4 Discussion

HCC has witnessed a steady increase in both incidence and mortality worldwide over the past few years (Oh and Jun, 2023; Qiu et al., 2024). Although notable progress in both diagnostic techniques, overall survival outcomes with HCC remain suboptimal in clinical settings. This phenomenon is largely attributable that HCC cases are associated with a substantial risk of postoperative recurrence and metastasis (Hanna et al., 2020; Yıldırım et al., 2023; Berardi et al., 2024). Thus, an urgent demand exists for the discovery of robust biomarkers, which could aid in the early detection of HCC and provide potential therapeutic targets, thereby improving clinical outcomes for patients with HCC.

We performed multidimensional data mining and integrative analyses of cholesterol metabolism-related genes, which revealed that TUBB2A is predominantly and highly expressed in hepatocytes, whereas its expression is relatively low in immune cell populations. Moreover, TUBB2A levels were consistently higher in tumor tissues, suggesting that it may exert its biological effects. ROC curve analysis further demonstrated that TUBB2A possesses appreciable prognostic predictive value, and the slight increase in AUC with longer follow-up supports its potential applicability in long-term survival prediction.

TUBB2A encodes the β-2A tubulin subunit, a key component of microtubules involved in maintaining cell morphology, mediating intracellular transport, and regulating mitotic spindle assembly and chromosome segregation (Sferra et al., 2018; Fukuyama et al., 2022; Di Pasquale et al., 2025). As an essential regulator of cytoskeletal dynamics, aberrant expression or dysfunction of TUBB2A is closely correlated with the dysregulation of cell proliferation, migration and invasion, and has been implicated in a variety of malignancies (Haram et al., 2008; Joerger et al., 2011; Li et al., 2024). Furthermore, altered expression or mutation of TUBB2A can affect the isotype composition and stability of microtubules, potentially modifying cellular responses to microtubule-stabilizing and destabilizing chemotherapeutic agents, such as taxanes and contributing to differential chemosensitivity among tumors (Ti et al., 2018; Muguruma et al., 2022; Chew and Cross, 2023; Prota et al., 2023; Wang et al., 2023a).

Our experimental findings further validated the relevance of TUBB2A in HCC. WB analysis and IHC staining assays confirmed that TUBB2A was elevated in HCC samples relative to adjacent non-tumorous regions. Clinically, elevated TUBB2A expression was associated with shorter median survival, higher AFP levels, and more advanced pathological stage. Given the single-center retrospective design of this study, collinearity among certain clinical variables and an uneven distribution of risk factors may have influenced their significance in multivariate analyses. Nonetheless, the overall suggests that TUBB2A holds promise as a biomarker for HCC diagnosis and prognostic evaluation, despite its translational potential in clinical practice needs to be validated in larger, multicenter, and prospective patient cohorts.

Sustained tumor cell proliferation is a central driver of cancer progression and therapeutic resistance (Vasan et al., 2019; Hanahan, 2022; Anand et al., 2023). Consistent with this concept, our studies demonstrated that TUBB2A actively contributes to malignant phenotypes in HCC. Overexpression of TUBB2A was found to enhance the proliferative, migratory, and invasive capacities of HCC cell lines, while targeted knockdown of this gene exerted contrasting functional outcomes. Consistent with our *in vitro* findings, a nude mouse xenograft model validated the tumor-promoting role of TUBB2A *in vivo*, which furnishes compelling functional evidence supporting its oncogenic properties in HCC.

In this study, GSEA revealed that high TUBB2A expression was significantly enriched in pathways related to cholesterol metabolism, fatty acid degradation, and steroid biosynthesis, suggesting its potential involvement in lipid metabolic reprogramming in HCC. In addition, the enrichment of cell cycle-related pathways, such as DNA replication and PI3K-Akt signaling, indicates that TUBB2A may promote tumor cell proliferation and survival. To explore the relationship between TUBB2A and cholesterol metabolism, we further assessed key regulators of cholesterol homeostasis, including HMGCR, LDLR, SREBP2, and CYP7A1. HMGCR is the rate-limiting enzyme for *de novo* cholesterol synthesis, LDLR mediates cellular uptake of extracellular cholesterol, and SREBP2 serves as a master transcriptional regulator coordinating cholesterol biosynthesis- and uptake-related programs. CYP7A1 is critical for hepatic cholesterol catabolism by converting cholesterol into bile acids. Notably, these genes showed consistent changes following TUBB2A perturbation, suggesting that TUBB2A may be linked to a broader rewiring of cholesterol homeostasis (Saito et al., 2023; Xiao et al., 2023; Cao and Liu, 2023; Chan et al., 2026).

A study reported that TUBB2B can affect cholesterol metabolism by regulating the HNF4A-CYP27A1 axis, thereby promoting HCC progression. This suggests that different tubulin isotypes may be involved in regulating cholesterol catabolic pathways and, in turn, influence tumor biological behaviors (Wang et al., 2023b). In addition, an integrative analysis of NAFLD-associated HCC identified TUBB as a key gene, indicating its potential diagnostic and prognostic value, and *in vitro* experiments showed that TUBB knockdown could suppress malignant phenotypes (Luo et al., 2025). Therefore, tubulin-related molecules may be associated with the development and progression of metabolically driven liver cancer. Combined with our GSEA results and the consistent changes of cholesterol homeostasis regulators following TUBB2A perturbation, we suggest that TUBB2A may be linked to cholesterol metabolic remodeling in HCC. However, the specific upstream regulators through which TUBB2A acts, and whether it truly alters cholesterol metabolic flux, still require further mechanistic studies for validation.

Of note, results from time-dependent ROC analysis indicated that AUC corresponding to TUBB2A in predicting 1-, 3-, and 5-year overall survival rates reached 0.587, 0.591, and 0.600. Although the AUC increased slightly with time, the values remained around 0.6 across all time points. According to commonly used clinical performance criteria, this indicates that TUBB2A alone has limited discriminatory capacity in distinguishing, future studies could incorporate other genes to develop a multi-gene combined predictive model.

Moreover, although this study has preliminarily explored the role of TUBB2A in HCC progression and its relationship with cholesterol metabolism, there are still some limitations. First, the study primarily relies on gene expression data and does not delve into the actual changes in cholesterol metabolism. Future research could incorporate lipidomics and proteomics to better understand the full scope of TUBB2A’s function. Second, while *in vitro* cell models help reveal basic mechanisms, they cannot fully reflect the complexity of the tumor microenvironment. Future studies should use animal models to further validate the role of TUBB2A in HCC. Lastly, future research should focus on investigating the interaction between TUBB2A and key cholesterol metabolism transcription factors to gain deeper insights into its molecular mechanisms.

In summary, TUBB2A is significantly upregulated in HCC tissues and demonstrates certain prognostic predictive capability. Functional experiments confirmed its role in promoting HCC cell proliferation, migration, and invasion. Although TUBB2A has limited predictive ability for HCC prognosis, its involvement in cholesterol metabolism reprogramming supports its potential as a biomarker. Future research should focus on exploring the molecular mechanisms underlying TUBB2A’s role in cholesterol metabolism regulation and evaluating its clinical potential as part of multi-marker models or as a therapeutic target for HCC.

## Data Availability

The original contributions presented in the study are included in the article/Supplementary Material, further inquiries can be directed to the corresponding authors.

## Supplementary material

SUPPLEMENTARY FIGURE S1 Expression differences of TUBB2A in the TCGA cohort and its prognostic predictive ability. **(A)** Scatter plot showing TUBB2A expression levels in liver cancer and normal liver tissues. **(B)** ROC curve evaluating the prognostic predictive performance of TUBB2A. **(C)** Volcano plot showing the distribution of differentially expressed genes between TUBB2A-high and TUBB2A-low groups in the TCGA-LIHC cohort. **(D)** KEGG pathway analysis showing significant enrichment in cholesterol metabolism, fatty acid degradation, and steroid biosynthesis in the TUBB2A-high group. **(E)** GSEA of the “cholesterol metabolic process” in the TCGA-LIHC dataset, showing significant enrichment. **(F)** Reactome pathway analysis identifying the enrichment of cell cycle progression, DNA replication, and PI3K-Akt signaling pathways in the TUBB2A-high group. **(G)** Hallmark GSEA showing enrichment in the G2/M checkpoint, E2F targets, and MYC targets, supporting a role for TUBB2A in cell cycle acceleration and tumor growth. **(H)** GOBP GSEA identifying enrichment in fatty acid metabolic processes, amino acid metabolic processes, and xenobiotic metabolic processes in the TUBB2A-high group. Note: *P ≤ 0.05; **P ≤ 0.01.

SUPPLEMENTARY FIGURE S2 mRNA and protein expression of TUBB2A **(A)** mRNA expression levels of TUBB2A in HCC cell lines. **(B)** Western blot bands and quantitative analysis of TUBB2A protein expression in HCC cell lines. Note: **P* ≤ 0.05; ***P* ≤ 0.01; ****P* ≤ 0.001; *****P* ≤ 0.0001.

SUPPLEMENTARY FIGURE S3 Validation of knockdown efficiency in HCC cell lines **(A)** Knockdown efficiency of TUBB2A in Hep3B cells confirmed by RT-qPCR. **(B)** Western blot bands and quantitative analysis demonstrating TUBB2A knockdown efficiency in Hep3B cells. Note: NC group, negative control; si group, TUBB2A knockdown. ***P* ≤ 0.01; ****P* ≤ 0.001; *****P* ≤ 0.0001.

SUPPLEMENTARY FIGURE S4 Validation of TUBB2A overexpression efficiency in LM3 cells **(A)** Overexpression efficiency of TUBB2A in LM3 cells confirmed by RT-qPCR. **(B)** Western blot bands and quantitative analysis validating TUBB2A overexpression in LM3 cells. Note: NC group, negative control group; OE group, TUBB2A overexpression group. **P* ≤ 0.05; ***P* ≤ 0.01.

SUPPLEMENTARY FIGURE S5 Validation of TUBB2A knockdown efficiency in Hep3B cells. **(A)** Relative TUBB2A mRNA expression levels in Hep3B cells transfected with shTUBB2A or NC measured by RT-qPCR. **(B)** Representative Western blot images (left) and quantitative analysis (right) of TUBB2A protein expression in Hep3B cells transfected with shTUBB2A or NC. Note: NC, negative control; sh, short hairpin RNA. **P* ≤ 0.05; *****P* ≤ 0.0001.

SUPPLEMENTARY FIGURE S6 RT-qPCR analysis of key cholesterol homeostasis regulators. **(A)** Relative mRNA expression levels of HMGCR, LDLR, SREBP2, and CYP7A1 in Hep3B cells transfected with siTUBB2A or NC. **(B)** Relative mRNA expression levels of HMGCR, LDLR, SREBP2, and CYP7A1 in LM3 cells transfected with a TUBB2A OE or NC. Note: NC, negative control; si, TUBB2A knockdown; OE, TUBB2A overexpression. **P* ≤ 0.05; ***P* ≤ 0.01; ****P* ≤ 0.001; *****P* ≤ 0.0001.
