# Supplementary figures and images for: TUBB2A expression and its prognostic significance in hepatocellular carcinoma revealed by cholesterol-metabolism-related gene profiling
Source: Front Mol Biosci. 2026 Mar 16;13:1778506. doi: 10.3389/fmolb.2026.1778506 (PMC13033567; doi:10.3389/fmolb.2026.1778506)

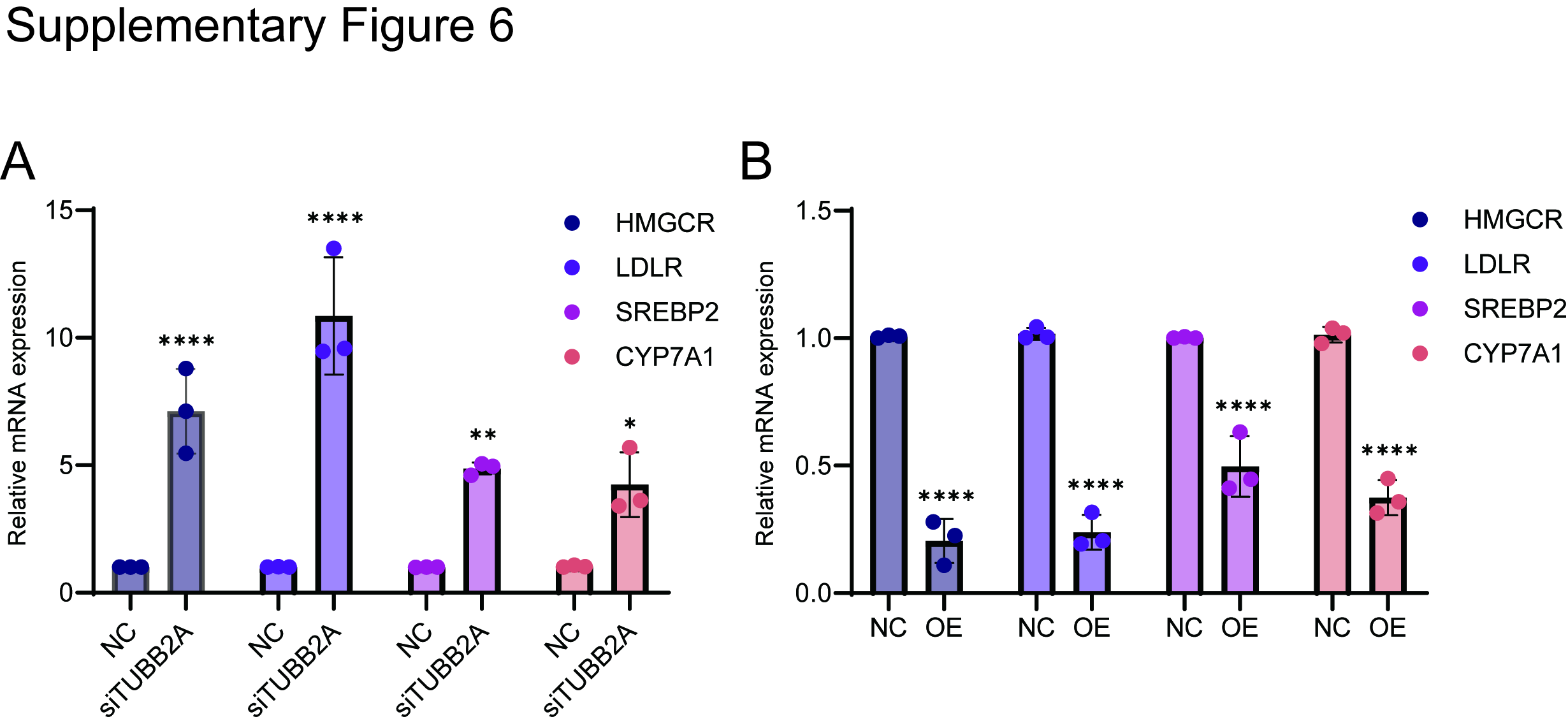

Supplement: Supplementary file 1 [file Image6.tif]

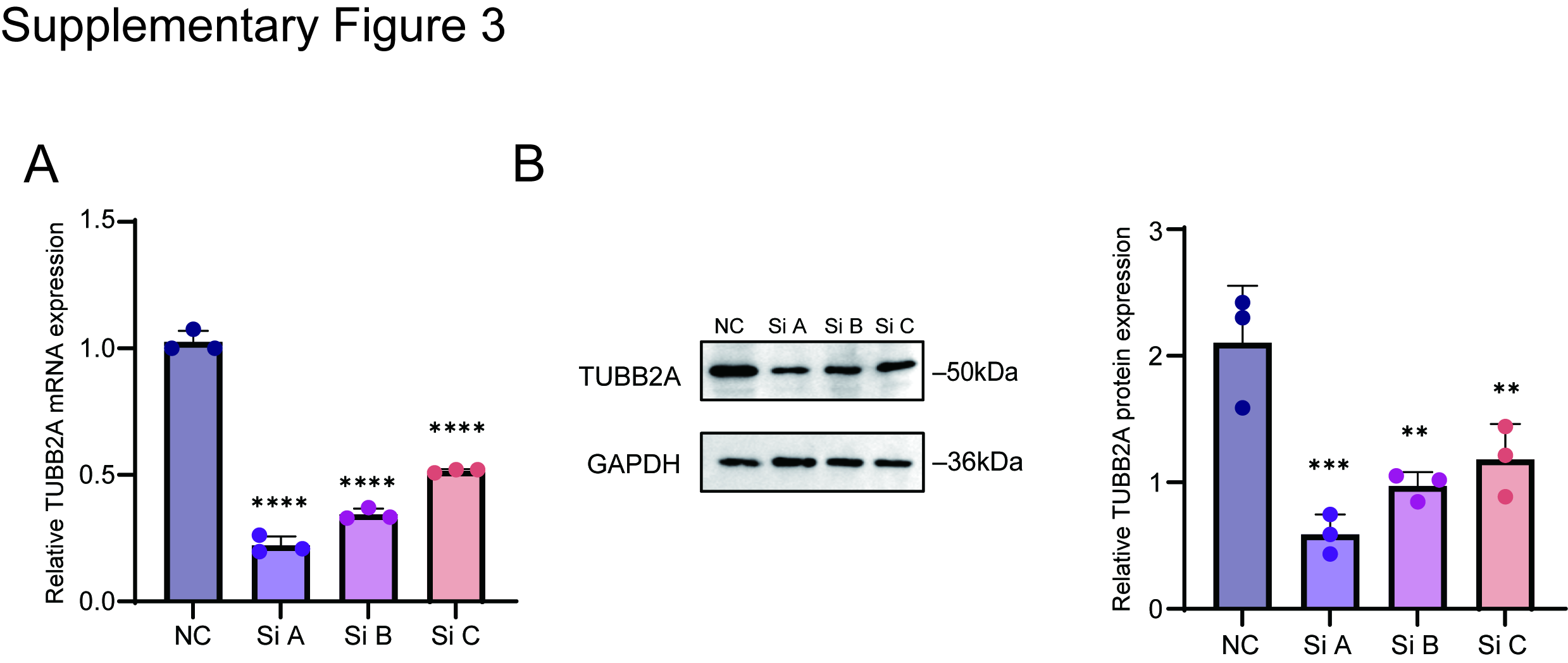

Supplement: Supplementary file 2 [file Image3.tif]

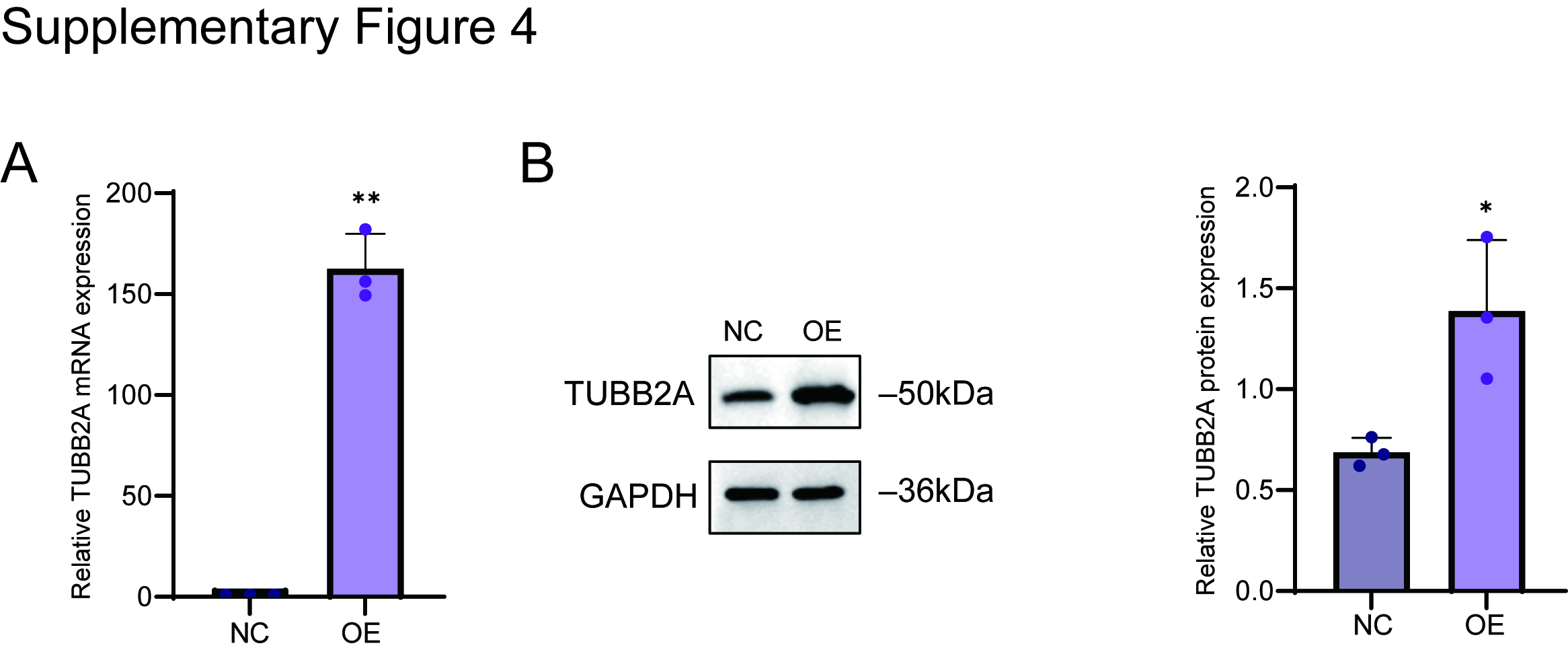

Supplement: Supplementary file 3 [file Image4.tif]

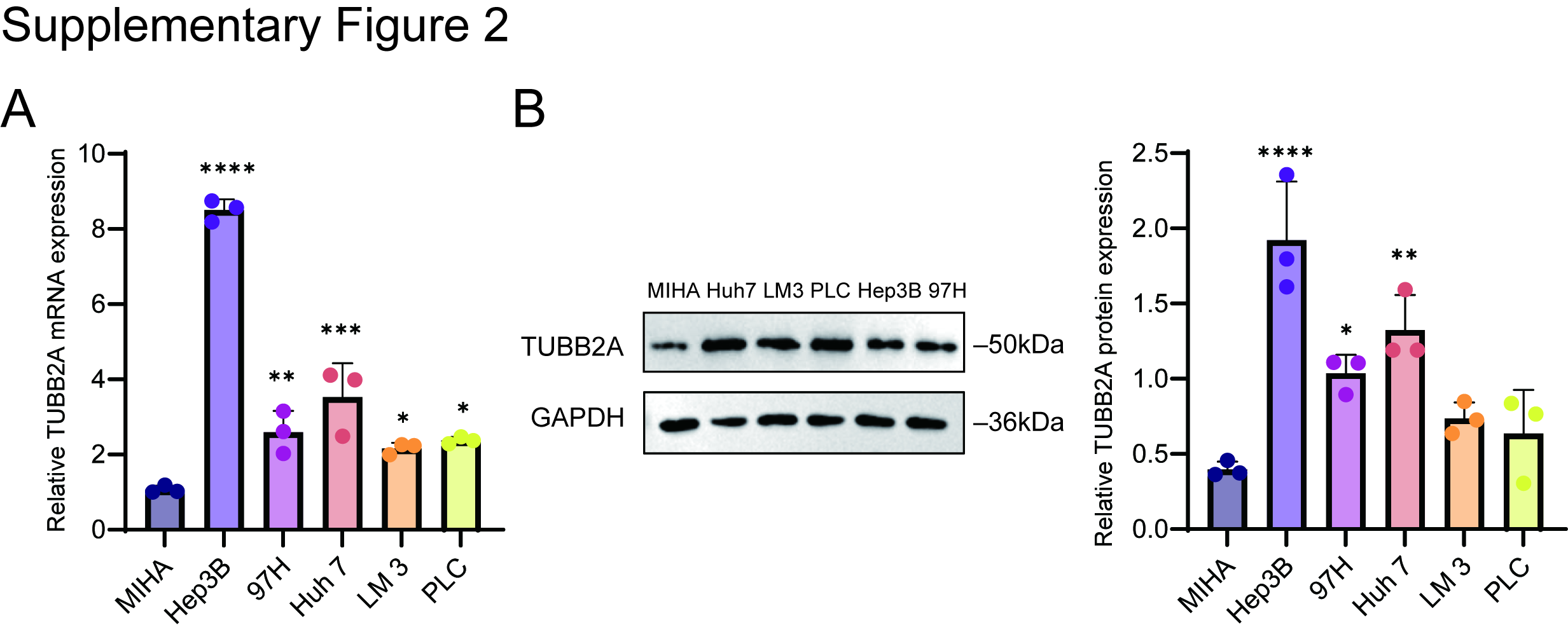

Supplement: Supplementary file 4 [file Image2.tif]

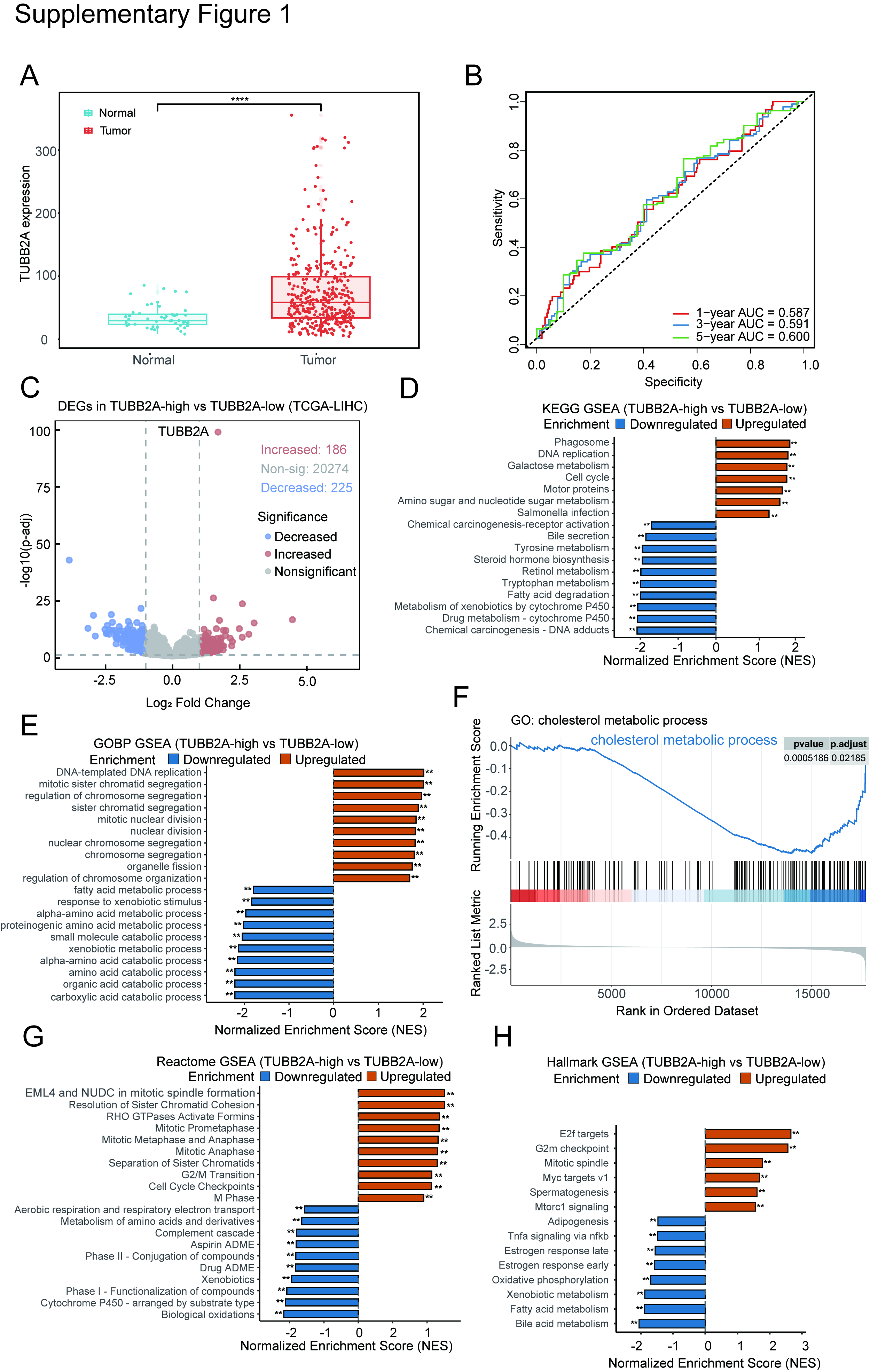

Supplement: Supplementary file 5 [file Image1.tif]

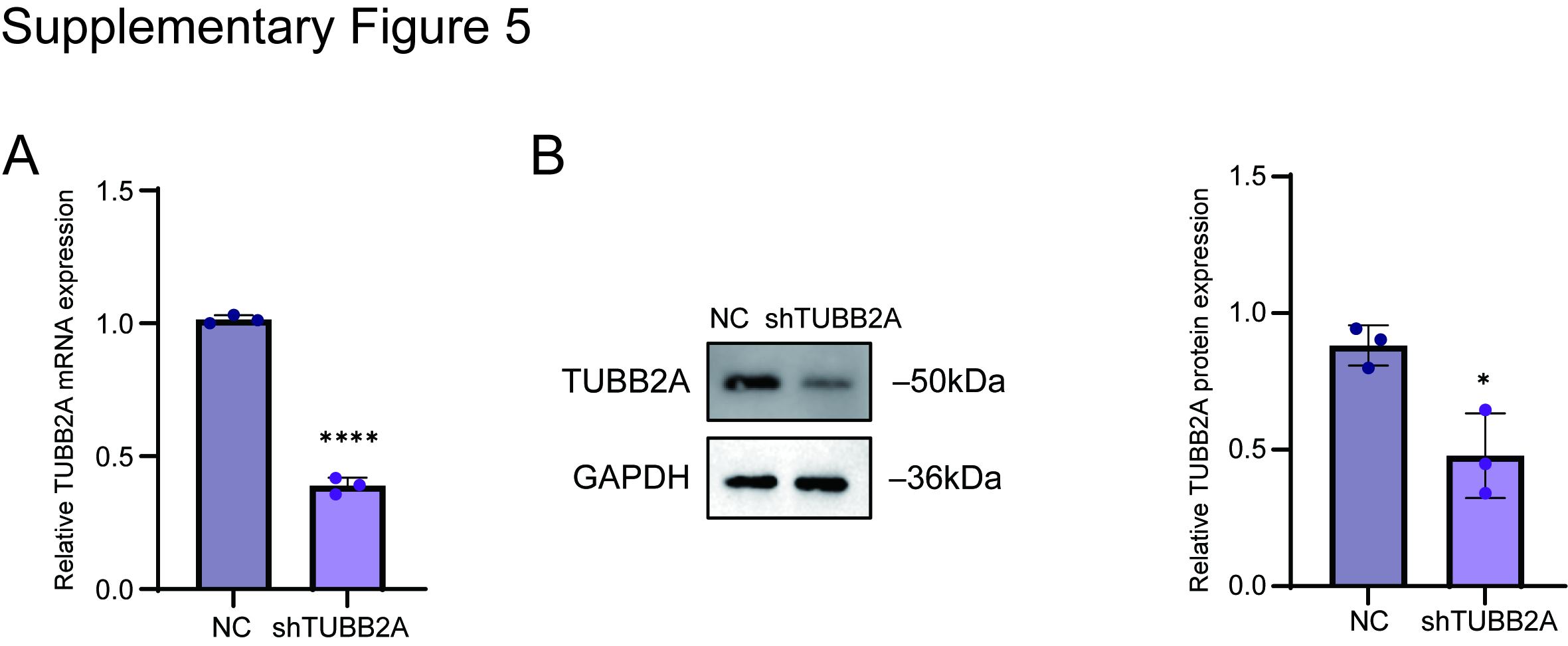

Supplement: Supplementary file 7 [file Image5.tif]
